# Patient complaints in general practice seen through the lens of professionalism: a retrospective observational study

**DOI:** 10.3399/BJGPO.2020.0168

**Published:** 2021-04-14

**Authors:** Pieter C Barnhoorn, Geurt TJM Essers, Vera Nierkens, Mattijs E Numans, Walther NKA van Mook, Anneke WM Kramer

**Affiliations:** 1 Department of Public Health and Primary Care, Leiden University Medical Center, Leiden, The Netherlands; 2 The Netherlands’ Network of the GP Specialty Training Institutes, Utrecht, The Netherlands; 3 Department of Intensive Care Medicine and Academy for Postgraduate Medical Training, Maastricht University Medical Centre, Maastricht, The Netherlands; 4 School of Health Professions Education, Maastricht University, Maastricht, The Netherlands

**Keywords:** general practice, after-hours care, patient complaints, retrospective observational studies, professionalism, professional development, professional identity formation

## Abstract

**Background:**

Professionalism is a key competence for physicians. Patient complaints provide a unique insight into patient expectations regarding professionalism. Research exploring the exact nature of patient complaints in general practice, especially focused on professionalism, is limited.

**Aim:**

To characterise patient complaints in primary care and to explore in more detail which issues with professionalism exist.

**Design & setting:**

A retrospective observational study in which all unsolicited patient complaints to a representative out-of-hours general practice (OOH GP) service provider in The Netherlands were analysed over a 10-year period (2009–2019).

**Method:**

Complaints were coded for general characteristics and thematically categorised using the CanMEDS Physician Competency Framework (CanMEDS) as sensitising concepts. Complaints categorised as professionalism were subdivided using open coding.

**Results:**

Out of 746 996 patient consultations (telephone, face-to-face, and home visits) 484 (0.065%) resulted in eligible complaint letters. The majority consisted of two or more complaints, resulting in 833 different complaints. Most complaints concerned GPs (80%); a minority (19%) assistants. Thirty-five per cent concerned perceived professionalism lapses of physicians. A rich diversity in the wording of professionalism lapses was found, where *'* *not being taken seriously* *'* was mentioned most often. Forty-five per cent related to medical expertise, such as missed diagnoses or unsuccessful clinical treatment. Nineteen per cent related to management problems, especially waiting times and access to care. Communication issues were only explicitly mentioned in 1% of the complaints.

**Conclusion:**

Most unsolicited patient complaints were related to clinical problems. A third, however, concerned professionalism issues. Not being taken seriously was the most frequent mentioned theme within the professionalism category.

## How this fits in

Research exploring the exact nature of patient complaints in general practice, especially focusing on professionalism, is limited. It was found that one-third of unsolicited patient complaints concerned professionalism issues. In addition, a rich diversity in the wording of professionalism lapses was found, where *'* *not being taken seriously* *'* was mentioned most often. By staying close to the words that patients use, the richness of the lessons that can be learnt from patient complaints can be preserved. These lessons provide important opportunities to improve GP care and GP training.

## Introduction

Professionalism is a key competence for all physicians.^1^ Lapses in physicians’ professionalism may affect health outcomes, therapeutic relationships, and the public’s perception and trust in the medical profession.^1–8^ Perceived professionalism lapses are part of patient complaints in all healthcare settings.^6,9–13^ GPs are especially vulnerable to patient complaints.^13–16^

Patient complaints provide unique and important insights into people’s expectations, especially as unsolicited complaints contain spontaneously provided information reflecting issues that are of high importance to patients and are not being captured otherwise.^5,6,10,17^ Complaints reflect patients’ expectations about provided care, especially concerning professionalism. Therefore, complaints are increasingly recognised as a potentially valuable source of information for improving healthcare quality.^6,7,9,18–20^ However, the exact relationship between complaints and quality of care is complex. Not all adverse events or all instances of patient dissatisfaction lead to complaints.^20,21^ Moreover, patient dissatisfaction may lead to complaints even when provided care has been exemplary.^20,21^ A further challenge in research on patient complaints is that professionalism issues may appear in many guises and can even be reflected in complaints if not explicitly mentioned.^4,7,9–11^ Moreover, when coding complaints using a standardised format, there is a danger of losing the richness of lessons that can be learnt from patient complaints.^10,22^ Research on patient complaints has the potential to address these challenges.

The exact nature of professionalism lapses often goes unnoticed because a universally agreed definition of professionalism is missing.^23–30^ Aspects of professionalism have been defined in terms of virtues (the good physician as a person of character) or behaviour (the good physician as a person who demonstrates competence).^27,29,31^ In the authors' view, CanMEDS, the General Medical Council (GMC) guidance, and the Ottawa Working Group on Professionalism provide sufficient direction for research on professionalism, as does research that describes and classifies unprofessional behaviours.^32–36^ However, what people expect of physicians regarding professionalism and what they consider lapses in professionalism need to be researched.

It should be acknowledged that professionalism can have different meanings in different contexts.^6,28,37–40^ Most research on what is perceived as (un)professional behaviour has hitherto been conducted in hospital settings.^2–7,41^ These studies found that most complaints are about medical, organisational, and communication issues as well as lapses in professionalism.^2,4–7,19^ Whether these findings are generalisable to settings outside the hospital is under-researched.^2–7,41^ Research focused on professional lapses in the GP context needs to be especially broadened and deepened, as previous research lacks the qualitative richness that patient complaints deserve.^13,19,42–45^ In OOH GP care, neither the patient nor the GP can benefit from a long-lasting relationship (in regular GP patient care this commonly exists); therefore, it is expected that professionalism lapses that may go unnoticed in regular GP care emerge more clearly in the OOH setting. The OOH context requires the utmost of a GP’s professionalism.^19,44,46,47^

Summarising the exact nature of patient complaints, in particular the perceived professional lapses, often remains enigmatic and unexplored, especially in the GP context. This study, therefore, aimed to answer the following research questions:

1. How can patient complaints in the GP setting be characterised?
2. What elements of physicians’ professionalism do patients address in these complaints?

## Method

To investigate the exact nature of patient complaints in OOH GP care, with a special focus on perceived professionalism lapses of physicians, a detailed content analysis was performed of original unsolicited patient complaints lodged at an OOH GP centre. The original patient complaints were used in order to stay close to the words that patients used, aiming to preserve the richness of lessons that can be learnt from patient complaints.

### Study context

The Dutch healthcare system is funded by a combination of tax contributions and a compulsory health insurance consisting of a per capita payment and fee-for-service. GPs are responsible for patients enlisted in their practice 24/7. On weekdays between 8.00 am and 5.00 pm primary medical health care is delivered by the GP practice. Outside office hours care is outsourced to the local OOH GP centres. Here, GPs answer emergency calls, offer consultations, and arrange home visits.^48^ The OOH GP cooperative in the present study (GP Services Rijnland) consists of three OOH GP care clinics. These clinics provide care for patients enlisted in GP practices in eight municipalities in both rural and (sub)urban areas, adding up to 325 000 inhabitants. These three clinics provide 75 000 calls, consultations, and home visits annually. If patients are dissatisfied with their care, they can lodge a complaint, either written, by email, telephone, or face to face, in a robust complaint system managed by a complaints officer.

### Study design and procedure

In this retrospective observational study, a content analysis was performed of all unsolicited healthcare complaints lodged at the OOH GP centre between 2009 and 2019, and all related relevant correspondence. For the purpose of this study, a complaint letter was defined as a letter (or transcript of a telephone or face-to-face encounter) that addresses one or more type of wrongdoing, offence, grievance, or resentment arising from the offered OOH GP service. A complaint was defined as every separately distinguishable type of wrongdoing, offence, grievance, or resentment that could be distilled from a complaint letter.

The original complaint letters were retrieved from storage, anonymised, and digitalised by the OOH GP complaints officer.

Excel software was used to organise the data. Descriptive statistics were used for quantitative analysis of the codes and categories. The STROBE guidelines were used in the conduct and reporting of this study.^49^ The study was performed in three steps.

The members of the research team were purposefully sampled to prevent blind spots in the analysis. All authors work as educational researchers and medical educators; four authors are clinicians, three are GPs. Walter NKA van Mook is an intensivist, Geurt TJM Essers is a psychologist, and Vera Nierkens is a health scientist specialised in health behaviour.

### Data analysis step 1: general characteristics

Where identifiable, the OOH GP complaints officer recorded: the sex and age of the patient whom the complaints concerned; whether the complainant was the patient in question, a relative, or another person involved (for example, the patient’s legal representative); to whom the complaint was directed (GP, GP resident, or the assistant); and how the complaint was submitted (by letter, email, telephone, or face to face).

### Data analysis step 2: themes

The first round of content coding of the anonymised original letters was open, inductive, and done with an iterative, constant comparison approach. Two authors analysed 25 randomly chosen transcripts and discussed their initial open coding. Distinct codes were assigned to each remark referring to different contents of the complaint. If a complaint letter concerned >1 aspect of care, each complaint was coded separately. Hereafter, one author analysed all 2009 and 2010 complaint letters (*n* = 90) and the open coding was discussed again. Two authors sought and found consensus on the axial coding scheme, which was then cross-checked with two other researchers. Subsequently, two authors performed selective coding, categorising the different codes into more abstract themes. Consensus on the themes was reached within the whole research team after two rounds of discussion. As the abstract themes paralleled the CanMEDS competencies, these competencies were used as sensitising concepts in a second round of deductive coding.^33^ It was decided to assign all complaints that could not clearly be categorised in one of the other six CanMEDS competencies to professionalism so as not to miss any authentic patient information.

Although data saturation was reached prior to finishing coding, all complaints were coded to ensure that the results accurately represented the frequencies and themes of the patient complaints.

### Data analysis step 3: professionalism

In order to answer research question 2, a deeper open analysis was conducted of the complaints coded as professionalism. It was also analysed whether these professionalism-related complaints stood on their own or were mentioned in combination with other complaints and vice versa.

## Results

Over the 10-year study period 746 996 patient consultations took place. The annual number varied between 70 853 (2013) and 84 410 (2018). These telephone contacts, face-to-face GP consultations, and home visits resulted in 493 complaint letters lodged. Three proved registrations of adverse events, five were addressed to healthcare professionals not in OOH GP care services, and one lacked detailed information, hampering further analysis. Consequently, nine complaint letters were excluded and 484 original complaint letters (concerning 0.065% of total consultations, annual percentage ranging from 0.059% to 0.161%) were analysed.

### General characteristics

The vast majority of the complaints (*n* = 362) were submitted by letter or email (75%), 116 by telephone (24%), and six in a face-to-face meeting (1%) (Table 1).

**Table 1.** General characteristics of complaints

Medium, *n* (*%*)	Complainant, *n* (*%*)	Aimed at, *n* (*%*)**^a^**	Sex, *n* (%)	Age, years, *n* (*%*)
Email or letter, 362 (75)	Patient, 198 (41)	GP, 389 (80)	Female, 259 (56)	0–18, 104 (31)
Telephone, 116 (24)	Parent, 147 (30)	Assistant, 90 (19)	Male, 206 (44)	19–64, 150 (45)
Face to face, 6 (1)	Partner, 63 (13)	Organisation, 49 (10)	Missing, 19 (4)	≥65, 77 (23)
	Child, 54 (11)	Resident, 6 (1)		Missing, 153 (32)
	Other, 22 (5)			
Total, 484 (100)	Total, 484 (100)	Total, 484 (100)	Total, 484 (100)	Total, 484 (100)

^a^Some complaints were aimed at more than one person.

Complaints were submitted by patients themselves (41%), their parents (30%), their partners (13%), or their children (11%). The remaining 5% were lodged by other relatives and colleagues. Most complaints were about GPs (80%). In 19% of complaints, the OOH GP care centre assistant was involved. Ten per cent of complaints were directed against the organisation of the OOH GP care centre. In six complaints (1%), the GP resident was explicitly mentioned as transgressing. The sex of the patient was not mentioned in 19 complaints (4%). Of the remaining 465 complaints, 259 (56%) related to female patients and 206 (44%) to male patients. The age of the patient was known in 331 complaint letters (68%). Of these, 104 were aged 0–18 years (31%), 150 were aged 19–64 years (45%), and 77 were aged ≥65 years (23%).

**Figure 1.**
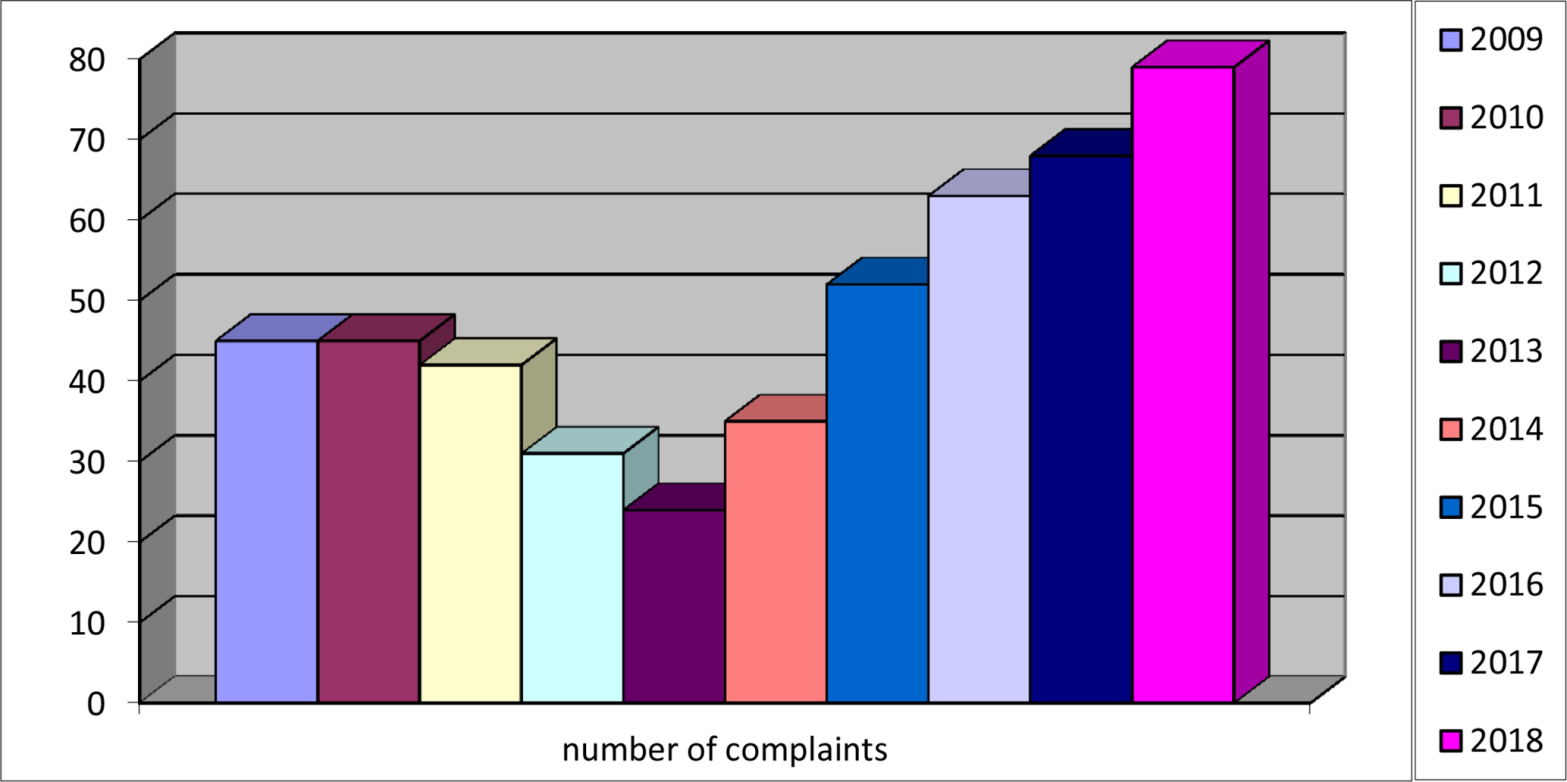
Number of complaints per year

After an initial decline in the number of complaints, an increase was observed from 24 complaints in 2013 to 79 complaints in 2018 (Figure 1). This absolute increase was accompanied by an increase in relative numbers.

Half of the complaint letters (49%) concerned one single complaint (data not shown). The remaining half concerned two or more complaints. In six cases there were up to five different complaints lodged at the same time. In total, out of the 484 analysed complaint letters, 833 different complaints could be distilled.

### Themes

Table 2 shows all complaint themes except those about professionalism. A total of 376 concerned medical expertise (45%), for example, missed diagnoses (predominantly missed fractures, myocardial infarction, and appendicitis), insufficient medical examination, poor or unsuccessful clinical treatment (such as incorrect placement of catheters or suboptimal stitching) and outdated, wrong, or absent advice. One hundred and nineteen complaints pertained to management issues (14%), for example, long waiting time for care, refusal to visit or consult, and finance and billing. Five complaints were solely about communication (1%), for example, not being called back. The remaining 333 complaints (40%) could not be clearly categorised in the above-mentioned CanMEDS competencies, that is, medical expert, manager and communicator, nor in the competencies collaborator, health advocate, or scholar, and were preliminary coded as professionalism. After analysing all 2009 and 2019 complaint letters (*n* = 90), no new themes emerged.

**Table 2.** Complaint themes except professionalism

Sensitising concept	Theme	***n* (%)**	Exemplary quotations (complaint identifier)
Medical expertise	Missed diagnosis	177 (21)	*'Eventually, the toe turned out to be broken after all.'* (1120)
			*'Because of persistent complaints, my own doctor later referred me to the cardiologist, who diagnosed myocardial infarction.'* (1003)
			*'The following day, my appendix was found to be inflamed and I had to have an operation immediately.'* (1431)
Medical expertise	Insufficient medical examination	99 (12)	*'He only felt with two fingers whether there was a temperature difference. Furthermore, he didn’t perform any physical examination.'* (1739)
			*'I was briefly examined and then dismissed.'* (1853)
Medical expertise	Poor or unsuccessful clinical treatment	71 (9)	*'However, placing the catheter had no effect.'* (1427)
			*'The anaesthetics did not go smoothly; the anaesthetic fluid came out through the wound and did not work.'* (1808)
Managing	Long waiting time for care	55 (7)	*'After three hours, there was still no doctor and the pain became unbearable for my wife.'* (1006)
Managing	Refusal to visit or consult	47 (6)	*'A doctor can never make a diagnosis over the phone! After repeatedly emphasising that it was really impossible to come to the clinic, the doctor even started a discussion.'* (1202)
			*'We had to wait over an hour in the waiting room.'* (1868)
Medical expertise	Outdated, wrong, or absent advice	29 (3)	*'Further advice was not given, so a restful sleep was not an option.'* (0944)
			*'When asked by my own doctor, this advice turned out to be incorrect.'* (1739)
Managing	Finance and billing	17 (2)	*'She received no advice during the phone call, on the contrary, the call was broken off for no reason at all. Because of this, we are unpleasantly surprised to have to pay an amount of 25 euros and request a remission of the amount.'* (1823)
Communication	Not called back	5 (1)	*'My brother was then informed that he would be called back by the doctor within 10 minutes about the situation. However, he has not been called back at all!'* (1701)

### Professionalism

The 333 complaints coded under ‘professionalism’ were explored in more detail. Of these 333 complaints, 290 were indeed about perceived lapses in physicians’ professionalism. The remaining 43 complaints were all found to be directed specifically against the organisation of the OOH GP care centre (for example, unhygienic working environment, insufficient or unclear signage, or non-medical advertising brochures in the waiting room) or the OOH GP care centre assistant (for example, unclear information about the clinic’s address and asking more information than necessary). These complaints were not investigated further, as they fell outside the scope of this study.

Patients articulated the perceived lapses in physicians’ professionalism in different terms. Examples included the following: not being taken seriously; being patronised; being unpleasantly spoken to; receiving inappropriate comments; perceiving a lack of empathy; perceiving the physician as being rushed; a physician who does not introduce himself or herself; not shaking hands; a physician who appears arrogant or uninterested; or displays physical harshness or unwanted intimacy. Table 3 shows explanatory quotes. The theme most frequently found within the professionalism category was 'not being taken seriously' (*n* = 88), mostly in regard to the health issue itself, the urgency, or the perception that one was seen as being overprotective.

**Table 3.** Explanatory quotes about complaints pertaining to professionalism

Themes	Explanatory quote
Not taken seriously	*'I am really angry that my complaint was not taken seriously.'* (1130)*'I am very angry that I was not taken seriously and have been dismissed as a hysterical person.'* (0913)*'Then, the doctor said: "and what was the urgent problem again?"'* (1108)*'What are you doing here? You have only had troubles for a few days now, and the OOH GP is only for emergency care.'* (1452)*'The doctor cannot find anything wrong and said: "You just have a cry-baby."'* (1136)
Patronised	*'Then, we were told that we were absolutely not allowed to consult the OOH GP for these complaints.'* (0919)
Spoken to unpleasantly	*'The doctor did not answer my questions, but barked at me.'* (1008)
Inappropriate comment	*'This comment was extremely out of place at that time.'* (1305)
Lack of empathy	*'She examined her with a total lack of empathy.'* (1105)
Rushed	*'I got the feeling that she was in a great hurry.'* (1311)
No introduction	*'The doctor did not introduce himself.'* (1754)
Not shaking hands	*'He did not shake my hand upon entering.'* (1106)
Arrogant	*'The doctor’s attitude was arrogant and disrespectful.'* (1413)
Uninterested	*'The doctor was sleepy, inattentive, and uninterested.'* (1763)
Physical harshness	*'The doctor was very hard-handed.'* (0901)
Unwanted intimacy	*'My daughter felt she was touched in an unpleasant way.'* (1435)

Of the 484 complaint letters, 213 contained complaints concerning lapses in professionalism, which in 87 (41%) cases was the only complaint. In 61 (29%) cases, a lapse in professionalism was combined with missed diagnoses, in 38 (18%) cases with insufficient medical examination, and in 19 (9%) cases with long waiting time for care.

## Discussion

### Summary

All patient complaint letters lodged at an OOH GP centre were thoroughly analysed with a special focus on perceived unprofessional behaviour of physicians. It was found that 746 996 OOH GP consultations over a 10-year period resulted in 484 complaint letters pertaining to healthcare professionals. Over one-third (35%) of the patient complaints concerned perceived lapses in physicians’ professionalism. A rich diversity in the wording of professionalism lapses was found, of which not being taken seriously was mentioned most often.

### Strengths and limitations

To the authors’ knowledge, the present study is the first to use content analysis of patient complaints in the context of primary care focused on GPs’ professionalism lapses. Moreover, the study period of a decade and the large number of complaint letters that could be analysed are unique. The OOH GP centre under study covers a large, diverse, and representative population of patients, which contributes greatly to generalisability of the results.

A few limitations should be noted. Notwithstanding the robust complaint system, not all adverse events or instances of patient dissatisfaction may lead to complaints.^20,21^ Moreover, complaints may be biased by negative health outcomes, as these outcomes may lead to patient dissatisfaction even when provided care has been exemplary.^20,21^ As in every analysis, information can get lost in translation to abstract themes. However, using a two-step analysis with both inductive and deductive methods, and multiple coding added to the rigour of this study.

### Comparison with existing literature

The data show a steady increase in patient complaints since 2013. This is in contrast with a recent study by Wallace *et al* on patient complaints in OOH GP, in which a relatively stable annual rate was seen of around 0.061% over a 5-year period, but is in line with other studies.^19,50,51^ Reasons for a potential increase, as mentioned in the literature, include a broader cultural change in society, including: changing expectations, nostalgia for a ‘golden age’ of health care, and the desire to raise grievances altruistically.^52,53^ This is in line with the many statements made in the complaint letters in the present study about the ‘desire for openness’ and the ‘hope that this won’t happen to others in the future’. The other general characteristics (medium, complainant, and aim) are consistent with the existing literature.^4,6,9,10,19^ This also applies to the frequency distribution that was found, with most complaints being about the medical expert role followed by complaints about professionalism and management.^5,7,10,13^

The results match well with Reader *et al*’s taxonomy for patient complaints and their ensuing Healthcare Complaints Analysis Tool (HCAT).^10,22^ Previous research using the HCAT for patient complaints in an OOH GP setting confirms the usability of this taxonomy in the (OOH) GP setting, although it is primarily based on research in hospital settings.^10,19,51^ However, the 290 complaints about perceived lapses in physicians’ professionalism could be placed in at least four categories of the HCAT (respect and patient rights, listening, communication, and quality).^10,22^ Therefore, a deeper analysis of patients’ rich vocabulary regarding professionalism was performed, which aimed to explore what people expect of physicians regarding professionalism and what they consider lapses in professionalism. The authors aimed to stay close to the words that patients used (not being taken seriously, being patronised, being unpleasantly spoken to, and so on) to avoid losing the essence of the complaint in the translation to more abstract predefined themes. This provided unique and important insights into patients’ expectations and their feelings about the provided care, especially concerning professionalism, which allows us to learn from these complaints.^4,6,7,9–12,19,20^

The percentage of what were considered lapses in professionalism (35%) is average and in line with the existing literature. Mattarozzi *et al* found relationship aspects to be the cause of complaint in 52.8% cases.^7^ Wofford *et al* found disrespect, with 36%, their most identified category.^4^ In their extensive review on 59 studies, reporting 88 069 patient complaints, Reader *et al* found that 29.1% related to healthcare staff—patient relationships. Contrarily, Schnitzer *et al* found that only a relative proportion of 9.3% of complaints were about the physician—patient relationship.^9^ However, it is thought the percentage of lapses in professionalism might even be higher because professionalism can be expressed via the performance of other competences.^54^ This could explain the relatively high percentage of combinations of competencies that were complained about in one complaint letter.

### Implications for research and practice

In line with most of the other literature on patient complaints, the results show that unmet expectations were a driver for many complaints.^5–7,11,13,19,41,44,55^ Therefore, GPs and future GPs have to be informed that they need to actively address patient expectations during consultations. They need to communicate about examination, treatment, potential complications, and prognosis.^19^

In postgraduate medical education and continuing medical education training, attention should be paid to the fact that professionalism lapses often occur and that these lapses can have a wide range of devastating consequences.^1–8^ By analysing patient complaints using CanMEDS, the authors want to facilitate the implementation in GP training. The findings of this study provide direction and underline the utter importance of (bi-directional) direct observation of residents by their supervisors in the OOH GP setting.^56^

Further research should focus on deeper analysis of complaints concerning professionalism, because perceived lapses in professionalism are frequently complained about but are articulated by patients in different ways. In-depth interviews are needed to further investigate the subtleties of how lapses in professionalism are perceived.^54^
